# The sorrow comes when I’m having moments of joy—experiences of parenting a live baby following a previous stillbirth: an interpretative phenomenological analysis

**DOI:** 10.3389/fpsyg.2024.1485278

**Published:** 2024-11-07

**Authors:** Hope Blocksidge, Alexander E. P. Heazell, Anja Wittkowski, Debbie M. Smith

**Affiliations:** ^1^School of Health Sciences, The University of Manchester, Manchester, United Kingdom; ^2^Greater Manchester Mental Health NHS Foundation Trust, Manchester, United Kingdom; ^3^Manchester Health Alliance Science Centre, University of Manchester, Manchester, United Kingdom; ^4^Saint Mary’s Hospital, Manchester University NHS Foundation Trust, Manchester, United Kingdom; ^5^Maternal and Fetal Health Research Centre, University of Manchester, Manchester, United Kingdom

**Keywords:** stillbirth, parenting, bonding, lived experience, bereavement, mothers, fathers

## Abstract

Stillbirth can lead to complex and varied psychological outcomes for parents. Many choose to have another pregnancy following a stillbirth; however, little is known about the experience of parenting and bonding with the subsequent baby. Couples, who were the biological parents of a stillborn baby and at least one subsequent live baby aged under five, were recruited and interviewed individually. Data were analysed using interpretative phrenomenological analysis. Twelve individual interviews (of six couples) were conducted and four themes with nine subthemes were developed. Theme 1 “*Back to the starting line: pregnancy as a means to an end*” captured parents’ desire to bring a live baby home with pregnancy being experienced alongside fear, trauma, and grief. Theme 2 “*Reality hits*” encapsulated the experience of arriving home and feeling overwhelmed by the demands of a new-born baby. Theme 3 “*Being a living and loss parent*” captured the experience of being a parent to both a living and non-living baby with conflicting emotions. Theme 4 “*Protection:* ‘*I need him there next to me, so I know he’s alive*’” represented the fear some parents felt when parenting their live baby and included parents’ strategies to manage this anxiety. This study presents novel insight into the complexities of being a parent to a stillborn baby in tandem with a live baby, with difficulties arising in bonding, and managing emotional distress linked to trauma and grief. Potential implications for care includes a need for increased training for professionals providing postnatal care.

## Graphical abstract

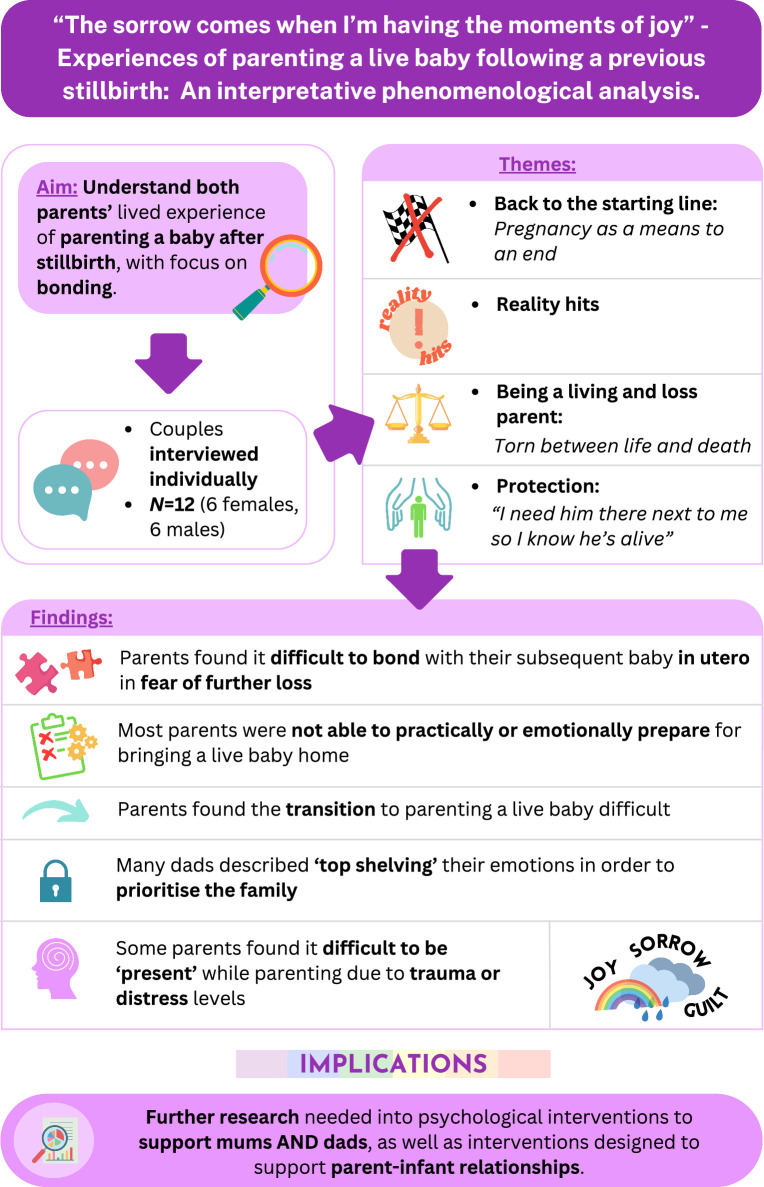

## 1 Introduction

In the UK, in 2021 there were 2,473 stillbirths (ONS, 2023). Stillbirth can lead to great sorrow for families, with not only the loss of dreams, expectations, and plans of the future that their baby signified, but also significant economic or psychosocial consequences for families (Due et al., 2017; Heazell et al., 2016). Whilst psychological distress follows all types of perinatal death, stillbirth has shown to lead to significantly higher levels of posttraumatic growth, post-traumatic stress symptoms, grief, and rumination levels compared to those who experience early miscarriage (Ryninks et al., 2022). In relation to the birth itself, couples can also experience increased levels of birth trauma with some parents later developing symptoms of PSTD (Campbell-Jackson et al., 2014). Women have described their experiences of stillbirth leading to various emotions including guilt, shame, anxiety, as well as physical effects such as chronic pain and fatigue (Heazell et al., 2016). Longer term outcomes of perinatal death are also extensive, including depression, anxiety, OCD behaviours, substance use, PTSD, and marital conflict (Cacciatore, 2013). Whilst the majority of research has centred around the mother, there is also growing evidence of negative psychological outcomes for partners post stillbirth with research from Jones et al. (2019) highlighting the complex position of being a supportive partner as well as managing their own grief. Additionally, the psychosocial impact for fathers has been identified to include grief suppression (avoidance), increased substance use, and employment difficulties or financial debt (Heazell et al., 2016).

Although there have been studies of women’s experiences of pregnancies after stillbirth, there are few studies which explore the longer-term experiences of parenting and bonding after the birth of a live baby. Understanding this experience of pregnancy and parenting is crucial due to 86% of women who have a stillborn baby become pregnant within 18 months of their loss (Mills et al., 2014). Using IPA, Üstündağ-Budak et al. (2015) explored six mothers’ experiences of stillbirth and their subsequent relationships with their live children. The notion of betrayal (of the stillborn baby) appeared prominent for participants with mothers reporting heightened anxiety when pregnant, parenting and caring for the subsequent infant. This research shines a light on the importance of parent-infant relationships; however, it lacks a fathers’ voice. Phenlan et al. (2013) study exploring six mothers’ experience of pregnancy after stillbirth (p. 65) also identified difficult feelings towards subsequent children with some mothers reporting feeling “standoffish” towards the subsequent child. Interestingly, Phenlan et al. (2013) findings also offered a different view of how some parents cherished their second pregnancy and adopted a highly involved parenting role, which highlights the individual differences in approaches to parenting following stillbirth.

When exploring the relationship between the infant and caregiver, the terms bonding and attachment are central. In the postnatal period, bonding is understood as the emotions and feelings that caregivers hold towards their infant, whereas attachment is the reciprocal relationship between the infant and the caregiver (Wittkowski et al., 2020). We know that the parental bonding process can lead to poorer attachment outcomes for the infant (Nordahl et al., 2020); however, less is known about father-to-infant bonding and how this, as well as the parenting journey, is affected by a previous stillbirth. The current study aimed to explore both the mother and fathers’ lived experience of parenting a baby after a stillbirth, with a focus on bonding.

## 2 Methods

### 2.1 Design and ethical approvals

This qualitative study aimed to interview up to six couples individually, utilizing Interpretative Phenomenological Analysis (IPA; Smith et al., 2021). Phenomenology as a theoretical framework is grounded in the assumption that human beings make sense of the world through personal experience (Husserl, 2012). Human experience is a highly complex notion and intertwined with culture, societal constructions and psychological experience, and thus, the way humans experience traumatic events can vastly differ (Alhazmi and Kaufmann, 2022). IPA aims to gain a detailed insight into specific and complex experiences, in this case, the experience of stillbirth, and allows for exploration of patterns and deviations with and across participant narratives (Murray and Wilde, 2020). Unlike other qualitative approaches, IPA’s background in phenomenology, hermeneutics and ideography, encompasses multiple theoretical positions (Smith et al., 2021). This method has been used in similar research contexts with similar sized samples (Campbell-Jackson et al., 2014; Üstündağ-Budak et al., 2015).

Ethical approval was obtained from the university’s research ethical committee (UREC; ref. 2021-12345-67890). Experts by experience were consulted during the research design process, in line with best practice UK legislative frameworks (HRA, 2023).

### 2.2 Participant inclusion and exclusion

Participants were eligible if both partners were the biological parents of at least one live baby after a self-reported previous stillbirth, using the UK definition. This live baby had to be the first live born for both partners and be under the age of five at the time of interview. Due to the focus in becoming a parent of a live baby following a previous stillbirth, the research team excluded couples who had a live baby prior to loss, which was in line with a qualitative exploration into parenting following stillbirth (Campbell-Jackson et al., 2014). Parents of more than one live baby post-stillbirth were included in the study as long as the eldest was under 5 years of age. The research team discussed the age of the liveborn baby at time of interview and concluded children up to the age of five would be included due to the importance of brain development in the first 5 years (Grantham-McGregor et al., 2007). Additionally, a child is likely to meet a number of developmental milestones over the first 5 years of life (Misirliyan et al., 2023), all of which impact the experience of parenting, and thus including this age felt appropriate to the research team. Both partners had to be proficient in English and give consent to take part in the study. Exclusion criteria included previous babies being born via surrogacy, current pregnancy, previous diagnosis/es of psychotic mental illness present at the time of stillbirth and/or live birth with no previous psychiatric hospital admissions. Other exclusions included current suicidal ideation.

### 2.3 Recruitment and study procedure

Participants who were in a couple relationship were recruited from social media via tweets and posts linked to a poster advertising the study with the lead researchers contact details. Participants were also recruited from charities sharing the study on their social media, including Tommy’s and Willow’s Rainbow Box. The research team continued to repost and reshare on a tri-weekly basis throughout recruitment for the study.

Participants contacted the lead researcher via email to express interest in the study. The lead researcher screened each person from the couple individually via email to ensure participants met the inclusion/exclusion criteria and had read the participant information sheet. Once screened, an interview on Microsoft Teams was arranged. Prior to interview, participants were sent a link to complete a short demographic questionnaire and consent form.

### 2.4 Topic guide development

In consultation with a patient and public involvement expert, the current literature and the research team, a semi-structured topic guide was developed. It was designed so that it could be used flexibly throughout the interview, with open-ended questions to explore areas most salient for participants who identified as mothers and fathers (Smith et al., 2021). The researcher conducted a pilot interview to assess its appropriateness. As there were no suggestions for major changes to the guide, the two participants of this couple that took part in the pilot study were included in the overall sample.

### 2.5 Data collection

Participants were asked to choose a pseudonym. In the event that participants did not assign themselves a pseudonym, the researcher gained their consent to assign them a pseudonym to maintain confidentiality. Identifiable information, such as locations, were omitted from transcripts. Consent was also gained verbally at the start of interview for the researcher to assign their live baby a pseudonym. At participants’ request, names of all stillborn children were retained, and this was approved by the institutional research ethics committee. Due to the importance of participants’ stillborn baby’s name being retained and heard in this study, the researcher acknowledged all names in the acknowledgements of this paper; however, due to participant confidentiality, real names were not used in quotes.

Demographic information from the demographic questionnaire was collected to contextualise individual narratives and the overall sample. To capture each individual’s narrative and due to prior research highlighting a number of different themes for partners experiencing perinatal death compared to the mother (Horstman et al., 2023; Azeez et al., 2022), participants were interviewed individually via Microsoft Teams. Following the interview, participants were debriefed and signposted to helplines for further support. Each participant was offered a £20 voucher for taking part. Transcripts were not returned to participants for comment or feedback due to time constraints.

### 2.6 Data analysis

All interviews were conducted, video recorded via the Microsoft Teams record function, and transcribed verbatim by the lead researcher. The lead researcher conducted interviews online at their home in a confidential area. The researcher’s handwritten notes made during interview were anonymised and scanned to an encrypted folder which was password protected.

Analysis followed IPA guidance by Smith et al. (2021) (see Table 1). The lead researcher analysed transcripts and developed experiential statements derived from the data, which evolved into Personal Experiential Themes (PETs) and Group Experiential Statements (GETs). The second researcher also individually analysed each transcript and both researchers met to ensure plausibility of the lead author’s themes. To ensure that themes were trustworthy and grounded in the data, the lead and second researcher had in-depth discussions following analysis of interviews. Each participant was analysed as an individual as opposed to a member of a couple. Any disagreements were discussed with reflection on researcher subjectivity and through re-reading the transcripts. These discussions were held mid-way through analysis and at the end. The discussions ensured that interpretation of themes was consistent with participant stories, thus protecting against data being influenced by researcher positioning (Smith et al., 2021). Finally, the whole research team reviewed and approved the final interpretations and written analysis.

**Table 1:** IPA process guided by Smith et al. (2021).

Phase		Description
Starting with the first case: reading and re- reading		Transcripts were independently read and reread by two researchers to allow for immersion in the data. Alongside this process, the lead researcher kept a notebook of powerful recollections and initial or striking observations about the transcript.
Exploratory noting		Exploratory comments were recorded alongside transcripts which examined semantic content and language use. Initial comments stayed close to the participant’s explicit meaning, from which the researchers began to identify how a participant talks about or understands a specific concept. Later comments attended to pronoun use, laughter, repetition, tone or degree of fluency. Furthermore, later conceptual comments took a more interrogative form in which the researcher began to ask questions of the data.
Constructing experiential statements		The researcher consolidated and crystallised thoughts by developing experiential statements. They began working with exploratory notes rather than the transcript itself to maintain the complexity of the transcript whilst reducing the volume of detail.
Searching for connections across experiential statements		Looking through the experiential statements, the researcher explored how they may fit together. Throughout this process the researcher returned to their recollections of the interview to ensure the clusters reflect the participants’ experiences.
Naming, consolidating, and organising personal experiential themes (PETS)		The researcher noted PETS for each participant. Those that are of highest-level organisation were noted in bold uppercase with subthemes written in lower case bold. Page numbers are noted with key words or phrases in order to contribute to an evidence trail.
Continuing the individual analysis of other cases		The above steps are repeated for each participant. Each participant and transcript are treated as a complete universe of inquiry.
Working with PET to develop group experiential themes across cases		The researcher identified patterns of similarity and differences across the PET that were generated in previous steps, to create a set of GETs.

### 2.7 Reflexivity, trustworthiness, and rigour

The lead researcher was a woman with several years’ experience of working psychologically with children and adults in various settings. At the time of conducting this study, she was practicing as a trainee clinical psychologist. All participants were aware the research was being conducted as part of a doctoral thesis. The second author is a practicing obstetrician and experienced researcher into causes and consequences of perinatal death. The third author is an academic and clinical psychologist with an interest in parenting and expertise in perinatal mental health. The last author was an academic psychologist specialising in health psychology research with an interest in parenting. A professional clinical relationship existed between one researcher (AH) and participants for some of the couples; however, this did not impact screening or the interview process due to two researchers being involved in recruitment. AH played no role in interviewing participants or the initial data analysis. All researchers had familial experience of pregnancy loss. Three researchers were also parents to living children. In supervision, researchers were mindful to ensure individual subjective positions did not influence interpretive accounts that were not reflected in the parents’ narratives. In order to achieve a subjective position, the lead researcher closely considered their positionality in relation to the work and continually reflected as to whether they stood as an outsider to the research participants, or an insider to the clinical area of work (Tuffour, 2017; Holmes, 2020).

## 3 Findings

### 3.1 Participant characteristics

Twelve biological parents (six mothers and six fathers) participated. Seven other individuals expressed interest in taking part but could not because they were pregnant at time of interview (*n* = 1), did not meeting the UK definition of stillbirth (*n* = 1) or did not respond to our screening email for participation (*n* = 5).

Participants were aged between 33 and 51. The majority of participants described themselves as White British (*n* = 9) with one participant describing themselves as Black British, one as Mixed White and Asian, and one as Other (Jamaican, Chinese and Italian). Participants had between one (*n* = 8) and two living children (*n* = 4). These children were between 3 and 48 months. Time between stillbirth and live birth ranged from 14 months to 36 months. Six individuals, equating to three couples were married (*n* = 6) with the other six individuals equating to three couples cohabiting (*n* = 6). Most participants reported no pregnancies between births (*n* = 10) with one mother reporting one miscarriage in between stillbirth and live birth (*n* = 1). Interviews took place between August and September 2023 and lasted between 76 min and 123 min. In two interviews, babies were present for feeding during the interview process.

### 3.2 Themes

Four Group Experiential Themes with subthemes were developed: (1) *Back to the starting line: pregnancy as a means to an end*, (2) *Reality hits*, (3) *Being a living and loss parent*, and (4) *Protection: “I need him there next to me, so I know he’s alive”* (Table 2).

**Table 2:** List of themes and subthemes.

	Group experiential theme (GETS)	Subthemes
1.	Back to the starting line: pregnancy as a means to an end	1.1.“Desperately, desperately wanted to bring our baby home” 1.2. Getting through: “we’ll deal with it after”
2.	Reality hits	2.1. Reality shock: the perfect baby 2.2. I must be grateful
3.	Being a living and loss parent	3.1. “I do parent them very, very differently” 3.2. Sorrow and joy
4.	Protection: “I need him there next to me, so I know he is alive.”	4.1. Safe or threat? 4.2. Protecting one’s own psychological wellbeing with support, in order to protect baby

#### 3.2.1 Theme 1: Back to the starting line: pregnancy as a means to an end

The first theme signified the decision to become pregnant after a previous stillbirth and consisted of two subthemes. The majority of parents spoke of a desire to have a baby with some suggesting this became the main focus or goal following their stillbirth. Parents spoke of pregnancy as a means to an end. The pregnancy bubble had been burst and instead, parents described intense fear for their baby. Through their experiences and as a way to protect themselves from further hurt, parents described how they often referred to their baby in hypothetical terms during subsequent pregnancies, *if* they made it home. Due to fear of future loss, all parents spoke of a lack of fetal attachment in pregnancy, impacting on the connection with their baby. As a result of fear, some parents described how bonding behaviours only started after the birth, whilst others, predominantly mothers, spoke of the bonding process taking longer to develop over the postpartum period.

##### 3.2.1.1 Subtheme 1.1: “desperately, desperately wanted to bring our baby home”

Parents discussed the feeling of longing for another baby following the death of their previous baby. This feeling appeared to arise early on in their grief, with Hannah suggesting her motivation for trying for another baby early on was due to the emptiness she felt after being pregnant for 9 months and having no baby to care for at home. All parents described a pressure to keep their baby safe to ensure they would be born healthy and able to go home with this sense of responsibility appearing particularly prominent for mothers, due to their ability to detect and monitor fetal movement:

“*I probably felt a sense of pressure as well, a little bit almost like I need to get this baby here safely*” (Ada).

Mothers spoke of their experience of fetal movement as having a medical focus to ensure survival, rather than using movement as an opportunity to interact with baby. The pregnancy journey was often described as pressurising and of high personal risk. Parents were all aware of the very real possibility of further heartbreak, and described being hypervigilant to any threat that indicated a chance of further loss, particularly towards bodily sensations or movement. This hypervigilance was often reinforced by medical professionals asking about movements on first interaction, and mothers spoke of this becoming overwhelming or increasing the pressure they had put on themselves to detect changes of movements.

Parents reflected on their experience with a sense that their pregnancy and parenting bubble had been burst when their baby died, so they found it very hard after this to imagine, talk about, and prepare for parenting a live baby. Some couples would only refer to their baby with terms such as ““*god willing*…” with one mother articulating how she “*felt like I could not even use language that presumed she was going to be alive”* (Hannah).

All parents spoke of the fear and level of risk for further loss they felt during pregnancy with one parent describing how “the stakes were high” during subsequent pregnancy (Patrick). Often fathers would speak about pregnancy and the fear they felt during this time as secondary to the needs of their partner, with Patrick describing how he had to shut down emotions in order to support his wife:

“*I kind of had to top shelf quite a lot of things just for the sake of running a house and all that sort of thing*” (Patrick).

Avoiding one’s own emotion to protect or prioritise the mother was spoken of by all fathers, and many discussed how they were only able to think about their own psychological needs when their baby after stillbirth was of weaning age and older.

##### 3.2.1.2 Subtheme 1.2: getting through: “we’ll deal with it after”

Instead of preparing for parenting, parents spoke of the objective being to get through pregnancy with parenting or bonding being something to *“deal with after”* (Cadmus). The only time parents described a pause in their anxieties was at hospital after attending a scan, with a build-up of anxiety prior to the appointment. No parent explicitly expressed enjoyment during the pregnancy other than brief moments when being monitored:

“*I can honestly say I did not enjoy a single moment of it. Unless I was on a monitor or a scan machine*” (Jade).

The thought of a live baby was often too overwhelming or too scary to contemplate for all parents, due to the anxiety of further loss. India described the fear of building a relationship or bond with her live baby, particularly *in utero*, due to her past experiences:

“*Just did not feel like it was a safe thing to do… I was like, yeah, no. I’m… I’m not talking to this baby. I’m finding it very hard to, to bond with this baby*” (India).

Interestingly, all parents chose to find out the sex of their subsequent baby, despite some parents choosing not to in previous pregnancies. Parents described how knowing the baby’s sex removed any unnecessary uncertainty for them during the pregnancy. Uncertainty was spoke of as being very difficult to tolerate in pregnancy and often the cause of great anxiety, so finding out the sex appeared to be key in managing these difficult emotions.

Some parents, particularly fathers, spoke of how more frequent scans within *The Rainbow Clinic* for pregnancy following loss, enhanced their connection with their baby *in utero* compared to general maternity scans:

“*I felt much more of a connection to her, but never looked at anything other than her being delivered safe and well*” (James).

Here James reflected on how this connection remained limited to thinking about the future only up to the birth and not beyond. It is also important to note that James used the word *connection* opposed to *bond* here, which could further emphasise the fear he experienced when thinking about building a relationship with the subsequent baby.

#### 3.2.2 Theme 2: Reality hits

This theme described the experience of arriving home with a live baby with little or no prior preparation for parenting. As aforementioned, parents were hesitant, or for some, were unable to plan for a live baby, and thus, some parents reported feeling overwhelmed with the reality of bringing a live baby home. Nearly all mothers described the complexities of transitioning from parenting a dead baby to a live baby, with a sense of shock around how challenging the experience of parenting a live baby can be. Feelings such as guilt, or pressure to be grateful for their subsequent baby were prominent within this theme. Parents, including fathers, described guilt as multifaceted; guilt would be triggered by finding the experience of caring for a live baby difficult or frustrating, and further triggered by having less time to dedicate to their stillborn baby. Managing the demands of both babies appeared a balancing act with parents noting that this felt like an ongoing process throughout every stage of parenting. This theme is comprised of two group experiential subthemes.

##### 3.2.2.1 Subtheme 2.1: reality shock: the perfect baby

Through their experience of stillbirth, some parents spoke of a romanticised view of parenting during and following the birth of their stillborn baby, with many dreams or hopes for that baby remaining in thought or imagination. One father explained how his wife viewed their stillborn baby as *“the perfect baby who could do no wrong” (David).*

Mothers including Ada, India, Jade and Hannah described bringing a live baby home as a *reality shock*. The thought of parenting had always been something too scary to consider, so when returning home, parents described feeling overwhelmed by the physical and emotional demands of a live baby. Other parents echoed the fear of planning for parenting, with one parent describing how she continued to find it difficult to plan or adjust to upcoming developmental stages or milestones due to a lack of psychological preparation:

*“I think my ability to think ahead for him is very limited… I cannot look ahead and plan ahead in the same way that I expect I would have been able to do for a different child*” (India).

##### 3.2.2.2 Subtheme 2.2: I must be grateful

The feeling of finding parenting difficult often triggered feelings of guilt with all parents describing an internal pressure to be “grateful” for their live baby:

“*It felt like a really difficult time of life, but then again, with that comes the guilt of well, you wanted these babies, and you have got them here safely and here they are… I almost did not want to be ungrateful for them being here safely*” (Ada).

Due to feelings of difficulty, pressures of feeling grateful, or feelings of guilt, some parents described an urge to overcompensate when parenting. Here, Hannah describes the mask she felt she had to put on in the early days of parenting whilst Tiana described overcompensating more for what she lost with her baby that died:

“*I was doing that originally where I felt I had to be on top form… And then the kids go to bed and I crash and feel really sad all the time… It’s learning about yourself, I suppose, and accepting that that’s OK.”* (Hannah).

Other parents described overcompensating for their own emotions such as guilt or anxiety:

*“I’m aware of that, I probably overcompensate with other things so he probably gets more in some ways*” (Sally).

Sally described how she has overcompensated in her parenting as a way of managing her own difficult emotions. She then explored how this urge could impact her response to behaviour in the future:

*“I do think that could possibly be a thing again like, I could let him get away with more than I should because I do not wanna… Yeah, I think that could be an issue, but I do know, I’m aware of it*” (Sally).

Sally named a very common worry in parenting; how to manage behaviour in toddlerhood. Sally noticed her own urge to allow boundaries to blur due to her difficult journey to parenthood. This urge to be lenient when dealing with behaviour highlights how an experience of loss could magnify certain worries or alter boundaries when parenting young children.

#### 3.2.3 Theme 3: Being a living and loss parent

This theme described how parents experience being a parent to both living and non-living babies. For all parents, keeping the memory of their baby alive was of utmost importance. Some parents described managing the demands of their liveborn and the relationship with their baby that died, well, and others described feeling torn between parenting both babies. This demand between life and death led parents to feel guilt for prioritising one baby over the other. The feeling of sorrow was described to run in parallel with joy when parenting, which also contributed to parents’ feelings of guilt. This theme encapsulated three subthemes.

##### 3.2.3.1 Subtheme 3.1: “I do parent them very, very differently”

Many parents, particularly mothers, used the word “parent” in relation to their ongoing relationship with their stillborn baby. One mother described how she viewed parenting as continuing to foster her connection with her baby that died:

*“I obviously have to parent her very differently… how I parent her now is that I am pursuing the legal case… I’m doing things like this and other research studies with preeclampsia, going on the radio, and that’s how I can parent her… We also have her in her Bunny, as I said, and take her out and have her own family photos*” (Sally).

Both mothers and fathers described parenting their baby that died by raising money in their baby’s name, celebrating birthdays, writing names in cards, or marking occasions such as starting school. All parents spoke of acts that honour their baby’s memory. Several fathers spoke of a desire to support others in a peer support context. These acts of memorialising their baby appeared an important part of managing grief for parents, with many highlighting the importance of keeping the memory as well as the name of their baby in the forefront of friends or families’ minds. One mother described how she viewed herself as a parent to her first born and how this differs from parenting her live baby:

*“I would say I’m a more honest parent with (Baby’s name), um if that’s even a thing… I’m more colloquial and more casual and more like he’s more my friend, rather than with Naomi I’m her mother…. So I do parent them very, very differently. Very differently*” (Jade).

Cadmus reflects on his experience of loss and how this has changed his view in parenting and added an additional appreciation for life, which acts now as his motivation to encourage his son to explore the world:

“*I do not have any fears for him to go out and have. No, no, no, I will not. I will not smother him in that respect. (Baby’s name) would not make him so valuable that I would not let him live. That’s not, if anything, I’d say, for me, it’s had the opposite reaction to that*” (Cadmus).

James echoed this view by suggesting losing their baby allowed him to appreciate life and has motivated him to make the choice to be more present, particularly in the early years:

“*I think I’ll probably a bit more… Present than I would have been*” (James).

The researcher was struck by the sense of parents being split whilst parenting with some describing guilt for not being able to allow time for their stillborn baby due to the demands of their live baby. This pressure to hold in mind two babies, then fuelled the feelings of guilt for not being present when parenting the live baby:

“*I feel guilty that I’m not always present*” (Sally).

The notion of being present was spoken about frequently by parents with one father describing how he struggled to remain present when looking at or interacting with his live baby:

“*I can tell that sort of looking at him, I’m thinking about her*” (Patrick).

Difficulty bonding with subsequent babies was spoken about frequently in relation to being present, mainly among mothers, with one mother stating how she “did not feel that rush of love” and remained “detached” from her live baby for months following birth (Jade).

##### 3.2.3.2 Subtheme 3.2: sorrow and joy

Some parents described a mixture of emotions when parenting, due to the loss they carried with them:

“*I do find that the sorrow comes when I’m having the moments of joy which is, which is sad, and I think that maybe will happen forever*.” (Tianna).

Sally described a sense of permanence to her sadness, which ran alongside the joy of parenting:

*“The sadness is still there. And I talk about both of them in that like they have got parallel paths and the grief, and the sadness is still there just as much as it was before. But then Jake’s path I am feeling you know, elements of happiness and joy and stuff as well*” (Sally).

Sally acknowledged that sorrow may accompany joy throughout life as a parent and allowing both her children to have parallel paths was important to her, in order to acknowledge her baby that died, as well as celebrate her son’s continued development. Other parents also mentioned how grief could arise when parenting. James described how he managed these emotions by dedicating time to sit with the memories or feelings of his daughter, at a time that he feels safe, in order for his own grief waves to pass:

*“A few people have said you need to be strong for –and I quickly interpreted being strong as ‘you need to you need to be in touch and deal with your own emotions’… something will trigger you… and then once the kids are in bed or at nursery or whatever, you can then sort of, sit back down and think about whatever it was that was coming out. Or just think about her for a bit”* (James).

#### 3.2.4 Theme 4: Protection: “I need him there next to me, so I know he is alive”

This theme encapsulated the fear or anxiety related to pregnancy and parenting for parents combined with the need to protect their baby from any harm. Parents spoke of the safety they felt at home; however, described intense anxiety when thinking about allowing others to look after baby, or allowing baby to explore the outside world. This theme includes descriptions of how parents managed their own anxiety or sense of risk, particularly in parenting strategies or behaviours, such as being over-cautious when their child is exploring, or limiting experiences that may hold risk. This theme was characterised by two subthemes.

##### 3.2.4.1 Subtheme 4.1: safe or threat?

When parenting their live baby, a parent’s sense of safety was key in maintaining control of anxious thoughts. There appeared to be a strong sense of safety at home; however, when parents began to think about leaving their baby with anyone in an environment outside of home, this quickly became threatening. Parents’ anxiety appeared to centre around fear of their baby dying and parents spoke of a strong need to protect their child from any possible threat:

“*So I think that she was like, 8 or 9 months before even for an hour, I’d handed her over to anybody, and I remember they were like, why do not you trust us… And I remember saying ‘cause she might die. Like, that’s how real it is. Like she might die’”* (Hannah).

For one parent, this sense of safety was only felt when she was physically with her son and spoke of co-sleeping to manage this anxiety: “*I need him there next to me, so I know he’s alive*.” Here, India describes the intense emotion attached to keeping her baby safe and highlights the difficulties of parenting in tandem with this emotion.

Many parents, including Hannah, Max and Jade described being very protective of their baby and saw others as not being able to fulfil the same protective role. Some parents spoke of giving lots of instructions for others if they were to look after their baby. For Hannah, this sense of protectiveness lessened with her second live baby.

*“I was really anxious about if they did not do everything that I said, exactly as I said, I found that really like anxiety provoking. And yeah, it’s been less intense with Jake for sure*” (Hannah).

Another coping strategy to manage anxious thoughts that parents described was seeking reassurance, either through regularly checking baby or waking at night to look at the baby monitor. This anxiety prompted some parents to learn basic lifesaving skills, either with a course or with YouTube videos.

“*So I always wake up. Look at it, see if she’s on her front, rush in, make sure she’s breathing”* (Joe).

Certain developmental stages were viewed by parents as anxiety inducing. Weening appeared particularly difficult for parents with nearly every parent noting their fear of choking. In one case, a parent described delaying their baby from moving to solid food from puree because of this anxiety:

“*She’s still on puree. And even when I give a textured food which is still puree, I’m terrified. So I know that I’m delaying her eating umm journey*” (Jade).

Other parents described feeling very safety conscious, and this being a new trait since the death of their baby:

“*I probably more than I ever thought, I probably have a bit of an irrational fear of I guess it’s like losing them and things like that and I’m a lot more safety conscious with them than I ever thought I would be…*” (Max).

##### 3.2.4.2 Subtheme 4.2: protecting one’s own psychological wellbeing with support, in order to protect baby

When parents discussed their anxieties, often their lived experience of support came into discussion. Ada, India, Jade and Hannah spoke of a lack of meaningful support postpartum with professionals such as health visitors making misjudged comments or being unhelpful with their advice:

“*The first couple were not that helpful… I did not feel like I was getting heard, being heard*” (Ada).

In absence of meaningful external support, some parents spoke of support from grandparents; however, due to many participants reporting relationships breakdown within their family as a result of stillbirth, many parents self-funded private services for psychological intervention:

“*I sought some private mental health support, we paid privately for a therapist… And thank goodness because I do not know what we would have done without it*” (Ada).

Psychological support that parents discussed was predominantly talking therapy, with the exception of four parents having EMDR therapy. Many parents spoke of immediate therapy offering a space to grieve in the aftermath of the loss of their baby. Whilst support further down the line allowed them to reflect on their own experiences and explore how these experiences may influence their parenting behaviours.

*“It made me realise my role within my family… You know where I fit within my family and how that worked. That was really useful*” (Hannah).

Parents also spoke about more practical support for parenting with one mother describing input she had which centred around attachment and connection with baby:

*“And yeah, we did like baby massage, which really helped with my bonding with him. And she called it NBAS… like watching him and his cues before he got to like being really distressed so that I could intervene and kind of just like getting to know him… I was kind of heightened all the time and I’m like panicking and waiting for something to go wrong, I wasn’t able to see those like subtleties, but like when I stood back and did, like chilled out a little bit, he was actually telling me things.*” (Sally).

The psychological input described in this quote increased this mother’s confidence in interacting with her baby, as well as supporting her to identify when her mental health could have been impacting on how present she is with her baby. Interestingly, the majority of those who spoke of benefit from psychological intervention were female, with fathers often reporting “shelving” their emotions in order to support their partner and children:

“*I have kind of top shelfed it, not because I’ve been like oh I’m not dealing with that and walked away, I kind of had to. And then it became convenient because of then the pregnancy and everything but now I need to sort of start unpicking it all and seeing what’s actually there”* (Patrick).

Patrick also spoke of benefit in peer support, which many parents reiterated. Support ranged from charity peer groups to networks such as Instagram. Mothers, including Jade and India often emphasised the benefits of social media in allowing them space to share their stillborn baby and interact with other bereaved parents. Hannah, in particular, spoke of peer support friends being her main emotional support whilst parenting her live baby.

“*So that gave me so much hope at the time that I was like, I am potentially not gonna feel this pain forever this bad”* (Hannah).

For some parents, there appeared to be a difference between the quality of support from friends who had similar lived experience compared to those who did not, with many describing how they feel different or isolated when interacting with other mums at baby groups or friends. Hannah also explained that her experiences of peer support gave her hope through early experiences of grief:

## 4 Discussion

This study explored experiences of being a parent to a live baby following a previous stillbirth, with a focus on bonding. Four themes provided a unique insight into the challenges of parenting and bonding with the subsequent baby to stillbirth, whilst retaining a meaningful connection with the baby that died.

During pregnancy with the subsequent baby to stillbirth, the thought of developing a connection with the baby was described by mothers and fathers as *scary* and many parents felt that assuming baby would come home alive would tempt fate. This sometimes led to a lack bond in pregnancy due to fears of further loss. Nevertheless, throughout the journey from stillbirth and throughout the subsequent pregnancy, parents appeared to develop expectations of parenting, with hopes and dreams for their family unit. This romanticised view of parenting was often contrasted to the difficult reality of parenting their subsequent new-born. This contrast between ideal and realistic parenting is a concept discussed in Sanders et al.’s (2023) qualitative study exploring the impact of idealistic expectations of parenthood (Sanders et al., 2023). Parents reported regret for a lack of preparation for parenting, with some suggesting the transition could be made easier if they had known what to expect or where to receive information or help for certain challenges.

When parenting throughout the developmental stages, parents described heightened anxiety, particularly around weaning. Anxiety was spoken of to either lead a parent to engage in reassurance behaviours such as checking baby is breathing, or restricting experiences that could be potentially risky, such as avoiding giving baby solid foods. Parents were often aware of their anxiety levels and some mothers described overcompensating for their own mental health while parenting their live baby, ensuring that their baby had every (and often more) opportunity to learn and grow in their first year. There was a strong desire by all parents to honour and keep their stillborn baby present in people’s minds. Parenting was described by mothers in particular, to occur from the point of conception and was also used as a verb to describe the continuing relationship between the stillborn baby and the parent. Using the term parent for the relationship between the parent and stillborn baby often created two parallel journeys as part of the parenting experience.

Existing literature exploring parental experiences of bonding with a baby in pregnancy following stillbirth support the current study’s finding. Firstly, Mehran et al. (2013) highlighted how previous perinatal loss can lead mothers to a sense differentiation between themselves and the foetus in the subsequent pregnancy, showing how dissociating from the pregnancy may be adaptive for parents in order self-protect against any future loss. In clinical practice, this dissociation may be observed as a tendency for parents to delay bonding with their baby *in utero* and professionals supporting parents in pregnancy should be mindful of keeping baby’s voice at the forefront of maternity care, to encourage the parent-infant connection. This lack of connection could partly explain difficulties in adjusting to parenting a live baby at home. We also know from Mihelic et al. (2016) that postnatal adjustment difficulties are reinforced by a lack of social support, combined with parental mental health difficulties. These factors may be particularly prevalent with parents of a live baby after loss, with parent’s often describing lack of faith in the support or a hesitancy to call on support, due to internal pressures to feel grateful for their live baby. Professionals working antenatally and postnatally with bereaved parents should be aware of this increased risk of adjustment difficulties and scaffold their support sensitively around both mothers and fathers.

The lack of bond between parent and baby appears to be central to the stories in this study. Research from Campbell-Jackson et al. (2014), highlighted similar bonding difficulties with a baby subsequent to perinatal loss and emphasised the necessity for professionals to normalise these difficulties in order to support both mothers and fathers (Campbell-Jackson et al., 2014). Whilst bonding difficulties might be expected, the importance of sensitive and attuned parenting when building early attachment relationships is crucial (Strathearn et al., 2009; Rogers et al., 2022). This is particularly relevant to the current study as parents reported feeling less present when parenting due to distress or previous trauma. Interestingly, some fathers contradicted this notion of being less present when parenting by suggesting their experience of stillbirth allowed them a new appreciation of life, and this often led to being more present, with some fathers changing jobs to allow them to spend more time with their family.

### 4.1 Implications

This study found that parenting a live baby following a previous stillbirth may present additional hurdles to the already difficult journey of parenting a new-born baby. In addition to bonding difficulties with the subsequent infant, the majority of parents spoke of becoming pregnant and parenting whilst dealing with traumatic experiences related to birth and loss of their stillborn baby. These trauma levels were described to lead parents to feel less present with their liveborn baby, as well as contributing to high parental anxiety throughout the early years of parenting their infant. Whilst there has been much effort to improve maternal mental health services with a shift towards trauma-informed care for new, expectant and bereaved mothers in the UK (NHS England, 2023; Blackpool Better Start, 2021), these services often cater for mothers exclusively. Our findings suggest that support is at present, inadequate, with many parents paying for private therapy. A scoping review of 23 studies from Hollins Martin and Reid (2023) highlighted the potential effectiveness of Cognitive Behavioural Therapy (CBT), Compassion Focussed Therapy (CFT), and grief counselling in managing trauma symptoms following perinatal bereavement. However, the interventions explored within this review were not delivered to a sample of mothers during pregnancy or following the birth of a live baby subsequent to stillbirth. Cortizo (2020) further discussed the effectiveness of delivering EMDR therapy post-loss and during subsequent pregnancy and highlighted how EMDR could improve a mother’s ability to process traumatic memories and support the mother to develop reflective functioning with their baby *in utero*, thus strengthening the bond between mother and infant. Future research is required to explore the use of all therapeutic approaches including CBT, CFT, grief counselling and EMDR, within the context of maternity services in NHS to explore effectiveness with mothers, as well as partners.

The current research highlights the need for psychological support for fathers, and further highlights the timing in which father’s access this support may need to be adjusted to when fathers feel able to explore emotions of experiences that they have previously shut down. In the current study, some fathers spoke of a need to know their partner was coping and their baby was home and safe, before embarking on psychological support.

Bonding difficulties as well as parental anxiety spoken of by parents highlight the need for parent infant services to scaffold their support around parents who have had a liveborn following loss. A good opportunity to begin delivering psychological support to families who are expecting a baby following a previous loss would be in services such as Rainbow Clinics (Graham et al., 2021). In appointments, maternity professionals should hold the baby’s voice in the forefront of their minds. We know reflective functioning has been highlighted to support postnatal bonding (Pazzagli et al., 2022) and therefore conversations during scans could aim to increase the parent’s reflective functioning of their baby. Professionals should also explore parental mental health support, as well as systemic interventions, including those supporting the parent-infant relationship; however, more research is required into evidence-based interventions for families following stillbirth.

The current study has also highlighted how parents often experienced a lack of meaningful or appropriate support in the postpartum period following their liveborn baby. The lack of helpful support and mistrust of services calls for further training or psychoeducation in the additional needs of families who parent a liveborn baby following loss. Findings also support the need for additional training for perinatal professionals in delivering compassionate, trauma informed care. As part of this, staff should be offered a space to reflect on their own experiences and self-compassion, in order to reduce burnout, and maximise the compassionate care they are able to deliver to their patients (Evans et al., 2023; Gilbert, 2014).

### 4.2 Strengths, limitations, and future research

This research had a strong sample consisting of 12 individual experiences of being a parent to a live baby following a previous stillbirth. Whilst this is a large sample for IPA, the analysis specifically focussed on 6 events due to individuals being in couple relationships. The researchers discussed sample size and decided that due to interviews concerning 6 events, this minimised possible implications of having a too large sample (Noon, 2018). The inclusion of couples is a particular strength with the fathers’ experience heard individually to the mothers. It might have been helpful to consider same sex couples within the present study; however, the research team were mindful of following the IPA principle of a homogenous sample and thus opted for heterosexual cisgender couples. This exclusion is a limitation of the study and calls for future IPA studies exploring the experiences of same sex couples parenting a live baby following a previous stillbirth. Another limitation of the current study is the demographics of the research team; all researchers were white European and held roles within healthcare. IPA centres upon the researcher’s interpretation of the participants experience (Smith et al., 2021), and thus a researcher’s own bias will be somewhat unavoidable in the research process. Researchers were aware of this bias and took aforementioned steps to address reflexivity; however, it remains a limitation that all researchers were of similar ethnocentric backgrounds.

Due to parents discussing the possible benefits of psychological support, future research is undoubtedly needed to build an evidence base for interventions designed to support families antenatally and postnatally following stillbirth.

## 5 Conclusion

This study was one of the first to explore the experiences of parenting a live baby following a previous stillbirth, with a focus on bonding. Findings highlight the additional pressures a parent might face when transitioning to becoming a parent of a live baby following a previous stillbirth. Parents described how they viewed parenting as involving both their stillborn baby and their live baby and this experience often impacted mental wellbeing by including emotions, such as guilt, sorrow, and joy as well as an internal pressure to be grateful for their live born. For some parents, fear of future loss fuelled bonding difficulties as well as parental anxiety; however, many parents spoke of the benefits of psychological therapy to manage their own mental health. The results of this study indicate a need for further research into psychological interventions designed to support families following previous stillbirth, alongside trauma-informed, compassionate maternity care.

## Data Availability

The raw data supporting the conclusions of this article will be made available by the authors, without undue reservation.

## References

[ref1] Alhazmi A. A. Kaufmann A. (2022). Phenomenological qualitative methods applied to the analysis of cross-cultural experience in novel educational social contexts. Front. Psychol. 13:785134. doi: 10.3389/fpsyg.2022.785134, PMID: 35548502 PMC9082033

[ref2] Azeez S. Obst K. L. Due C. Oxlad M. Middleton P. (2022). Overwhelming and unjust: a qualitative study of fathers’ experiences of grief following neonatal death. Death Stud. 46, 1443–1454. doi: 10.1080/07481187.2022.2030431, PMID: 35107411

[ref3] Blackpool Better Start. (2021). A good practice guide to support implementation of trauma-informed care in the perinatal period. Available at: https://democracy.blackpool.gov.uk/documents/s13827/Appendix%206a%20Better%20Start%20Strategy.pdf

[ref4] Cacciatore J. (2013). Psychological effects of stillbirth. Semin. Fetal Neonatal Med. 18, 76–82. doi: 10.1016/j.siny.2012.09.001 23040157

[ref5] Campbell-Jackson L. Bezance J. Horsch A. (2014). “A renewed sense of purpose”: mothers’ and fathers’ experience of having a child following a recent stillbirth. BMC Pregnancy Childbirth 14:423. doi: 10.1186/s12884-014-0423-x, PMID: 25522655 PMC4279693

[ref6] Cortizo R. (2020). Prenatal and perinatal EMDR therapy: early family intervention. J. EMDR Prac. Res. 14, 104–115. doi: 10.1891/EMDR-D-19-00046

[ref7] Due C. Chiarolli S. Riggs D. W. (2017). The impact of pregnancy loss on men’s health and wellbeing: a systematic review. BMC Pregnancy Childbirth 17, 380–393. doi: 10.1186/s12884-017-1560-9, PMID: 29141591 PMC5688642

[ref8] Evans D. Butterworth R. Atkins E. Chilvers R. Marsh A. Barr K.. (2023). The anatomy of compassion: courage, connection and safeness in perinatal practice. Infant 19, 88–92.

[ref9] Gilbert P. (2014). The origins and nature of compassion focused therapy. Br. J. Clin. Psychol. 53, 6–41. doi: 10.1111/bjc.12043 24588760

[ref10] Graham N. Stephens L. Heazell A. E. P. (2021). Care in pregnancies subsequent to stillbirth or perinatal death. Obstetr. Gynaecol. 23, 48–59. doi: 10.1111/tog.12708

[ref11] Grantham-McGregor S. Cheung Y. Cueto S. Glewwe P. Richter L. Strupp B. (2007). Developmental potential in the first 5 years for children in developing countries. Lancet 369, 60–70. doi: 10.1016/S0140-6736(07)60032-4, PMID: 17208643 PMC2270351

[ref12] Heazell A. E. P. Siassakos D. Blencowe H. Burden C. Bhutta Z. A. Cacciatore J.. (2016). Stillbirths: economic and psychosocial consequences. Lancet 387, 604–616. doi: 10.1016/S0140-6736(15)00836-3, PMID: 26794073

[ref13] Hollins Martin C. J. Reid K. (2023). A scoping review of therapies used to treat psychological trauma post perinatal bereavement. J. Reprod. Infant Psychol. 41, 582–598. doi: 10.1080/02646838.2021.2021477, PMID: 34989287

[ref14] Holmes A. G. D. (2020). Researcher positionality - a consideration of its influence and place in qualitative research - a new researcher guide. Shanlax Int. J. Educ. 8, 1–10. doi: 10.34293/education.v8i4.3232

[ref15] Horstman H. K. Morrison S. McBride M. C. Holman A. (2023). Memorable messages embedded in Men’s stories of miscarriage: extending communicated narrative sense-making and memorable message theorizing. Health Commun. 38, 742–752. doi: 10.1080/10410236.2021.1973718, PMID: 34503374

[ref9001] HRA. (2023). UK Policy Framework for Health and Social Care Research. Available at: https://www.hra.nhs.uk/planning-and-improving-research/policies-standards-legislation/uk-policy-framework-health-social-care-research/uk-policy-framework-health-and-social-care-research/

[ref9002] Husserl E. (2012). Ideas: General Introduction to Pure Phenomenology (1st ed.). Routledge. doi: 10.4324/9780203120330

[ref9003] Jones K. Robb M. Murphy S. Davies A. (2019). New Understandings of Fathers’ Experiences of Grief and Loss Following Stillbirth and Neonatal Death: A Scoping Review. Midwifery, 7:102531. doi: 10.1016/j.midw.2019.102531 31493675

[ref17] Mehran P. Simbar M. Shams J. Ramezani-Tehrani F. Nasiri N. (2013). History of perinatal loss and maternal–fetal attachment behaviors. Women Birth 26, 185–189. doi: 10.1016/j.wombi.2013.04.005, PMID: 23721683

[ref18] Mihelic M. Filus A. Morawaska A. (2016). Correlates of prenatal parenting expectations in new mothers: is better self-efficacy a potential target for preventing postnatal adjustment difficulties? Prev. Sci. 17, 949–959. doi: 10.1007/s11121-016-0682-z, PMID: 27438295

[ref19] Mills T. A. Ricklesford C. Cooke A. Heazell A. E. Whitworth M. Lavender T. (2014). Parents' experiences and expectations of care in pregnancy after stillbirth or neonatal death: a metasynthesis. BJOG Int. J. Obstetr. Gynaecol. 121, 943–950. doi: 10.1111/1471-0528.12656, PMID: 24589119

[ref20] Misirliyan S. S. Boehning A. P. Shah M. (2023). Development Milestones. St. Petersburg, FL: StatPearls Publishing. 32491450

[ref21] Murray C. D. Wilde D. J. (2020). Thinking about, doing and writing up research using interpretative phenomenological analysis. Handb. Theor. Methods Appl. Health Res. 12, 140–166. doi: 10.4337/9781785363214.00015

[ref22] Noon E. J. (2018). Interpretive phenomenological analysis: an appropriate methodology for educational research? J. Persp. Appl. Acad. Prac. 6, 75–83. doi: 10.14297/jpaap.v6i1.304

[ref23] Nordahl D. Rognmo K. Bohne A. Landsem I. P. Moe V. Wang C. E. A.. (2020). Adult attachment style and maternal-infant bonding: the indirect path of parenting stress. BMC Psychol. 8:58. doi: 10.1186/s40359-020-00424-2 32513300 PMC7278048

[ref9004] Office for National Statistics. (2023). Births in England and Wales - Office for National Statistics.

[ref24] Pazzagli C. Buratta L. Cenci G. Coletti E. Giuliani M. L. Mazzeschi C. (2022). Does parental reflective functioning mediate the associations between the maternal antenatal and postnatal bond with the child in a community sample? Int. J. Environ. Res. Public Health 19:6957. doi: 10.3390/ijerph19126957 35742206 PMC9222610

[ref25] Phenlan L. Daniluk J. Amundson N. Fairbrother N. (2013). *Experiences of pregnancy following stillbirth: A phenomenological inquiry.* Doctoral dissertation. Vancouver, BC: The University of British Columbia.

[ref26] Rogers C. R. Chen X. Kwon S. J. McElwain N. L. Telzer E. H. (2022). The role of early attachment and parental presence in adolescent behavioral and neurobiological regulation. Dev. Cogn. Neurosci. 53:101046. doi: 10.1016/j.dcn.2021.101046, PMID: 34954667 PMC8717427

[ref27] Ryninks K. Wilkinson-Tough M. Stacey S. Horsch A. (2022). Comparing posttraumatic growth in mothers after stillbirth or early miscarriage. Public Libr. Sci. One 17:e0271314. doi: 10.1371/journal.pone.0271314, PMID: 35939433 PMC9359608

[ref28] Sanders R. E. Lehmann J. Gardner F. (2023). Title: new parents’ idealistic expectations of parenthood: the impact of preconceived ideas. J. Fam. Issues 44, 850–871. doi: 10.1177/0192513X211055124

[ref29] Smith J. A. Flowers P. Larkin M. (2021). Interpretative phenomenological analysis: Theory, method and research. 2nd Edn. London: Sage Publications.

[ref31] Strathearn L. Fonagy P. Amico J. Montague P. R. (2009). Adult attachment predicts maternal brain and oxytocin response to infant cues. Neuropsychopharmacology 34, 2655–2666. doi: 10.1038/npp.2009.103, PMID: 19710635 PMC3041266

[ref32] Tuffour I. (2017). A critical overview of interpretative phenomenological analysis: a contemporary qualitative research approach. J. Health Care Commun. 02:52. doi: 10.4172/2472-1654.100093

[ref33] Üstündağ-Budak A. M. Larkin M. Harris G. Blissett J. (2015). Mothers' accounts of their stillbirth experiences and of their subsequent relationships with their living infant: an interpretative phenomenological analysis. BMC Pregnancy Childbirth 15:263. doi: 10.1186/s12884-015-0700-3, PMID: 26463456 PMC4604712

[ref34] Wittkowski A. Vatter S. Muhinyi A. Garrett C. Henderson M. (2020). Measuring bonding or attachment in the parent-infant-relationship: a systematic review of parent-report assessment measures, their psychometric properties and clinical utility. Clin. Psychol. Rev. 82:101906. doi: 10.1016/j.cpr.2020.101906, PMID: 32977111 PMC7695805

